# Quantifying transduction efficiencies of unmodified and tyrosine capsid mutant AAV vectors in vitro using two ocular cell lines

**Published:** 2011-04-29

**Authors:** Renee C. Ryals, Sanford L. Boye, Astra Dinculescu, William W. Hauswirth, Shannon E. Boye

**Affiliations:** Department of Ophthalmology, University of Florida, Gainesville, Fl

## Abstract

**Purpose:**

With the increasing number of retinal gene-based therapies and therapeutic constructs, in vitro bioassays characterizing vector transduction efficiency and quality are becoming increasingly important. Currently, in vitro assays quantifying vector transduction efficiency are performed predominantly for non-ocular tissues. A human retinal pigment epithelial cell line (ARPE19) and a mouse cone photoreceptor cell line, 661W, have been well characterized and are used for many retinal metabolism and biologic pathway studies. The purpose of this study is to quantify transduction efficiencies of a variety of self-complementary (sc) adeno-associated virus (AAV) vectors in these biologically relevant ocular cell lines using high-throughput fluorescence-activated cell sorting (FACS) analysis.

**Methods:**

ARPE19 and 661W cells were infected with sc-smCBA-mCherry packaged in unmodified AAV capsids or capsids containing single/multiple tyrosine-phenylalanine (Y-F) mutations at multiplicity of infections (MOIs) ranging from 100 to 10,000. Three days post infection fluorescent images verified mCherry expression. Following microscopy, FACS analysis was performed to quantify the number of positive cells and the mean intensity of mCherry fluorescence, the product of which is reported as transduction efficiency for each vector. The scAAV vectors containing cone-specific (sc-mCARpro-green fluorescent protein [GFP]), rod-specific (sc-MOPS500-eGFP), retinal pigment epithelium (RPE)-specific (sc-VMD2-GFP), or ubiquitous (sc-smCBA-GFP) promoters were used to infect both cell lines at an MOI of 10,000. Three days post infection, cells were immunostained with an antibody raised against GFP and imaged. Finally, based on our in vitro results, we tested a prediction of transduction efficiency in vivo.

**Results:**

Expression from unmodified scAAV1, scAAV2, scAAV5, and scAAV8 vectors was detectable by FACS in both ARPE19 and 661W cells, with scAAV1 and scAAV2 being the most efficient in both cell lines. scAAV5 showed moderate efficiency in both ARPE19 and 661W cells. scAAV8 was moderately efficient in 661W cells and was by comparison less so in ARPE19 cells; however, transduction was still apparent. scAAV9 performed poorly in both cell types. With some exceptions, the Y-F capsid mutations generally increased the efficiency of scAAV vector transduction, with the increasing number of mutated residues improving efficiency. Results for single scAAV1 and scAAV8 capsid mutants were mixed. In some cases, efficiency improved; in others, it was unchanged or marginally reduced. Retinal-specific promoters were also active in both cell lines, with the 661W cells showing a pattern consistent with the in vivo activity of the respective promoters tested. The prediction based on in vitro data that AAV2 sextuple Y-F mutants would show higher transduction efficiency in RPE relative to AAV2 triple Y-F capsid mutants was validated by evaluating the transduction characteristics of the two mutant vectors in mouse retina.

**Conclusions:**

Our results suggest that this rapid and quantifiable cell-based assay using two biologically relevant ocular cell lines will prove useful in screening and optimizing AAV vectors for application in retina-targeted gene therapies.

## Introduction

Advances in the development of recombinant adeno-associated virus (AAV) vectors together with recent successes in using AAVs in human clinical trials of retinal disease have resulted in an explosion in the use of AAVs for retina-targeted gene therapy. With the increase in number of characterized serotypes (both naturally occurring and engineered) and the availability of tissue-specific promoters, more emphasis has been placed on targeting specific cell types within the retina or even subclasses of cell types (e.g., cones versus all photoreceptors). Results of several ongoing phase I/II clinical trials for RPE65-Leber congenital amaurosis-2 (LCA2) indicate that AAV-mediated *Rpe65* delivery to the retinal pigment epithelium (RPE) is both safe and effective [1-4]. Several successful proof-of-concept studies involving mice, dogs, and monkeys have established that AAV-mediated gene replacement is also a feasible strategy for treating photoreceptor-specific disorders [5-8]. More specifically, because of their contribution to acute, daylight vision, much attention has been paid to disorders affecting cone photoreceptors. As with LCA2, the successful clinical application of vectors to treat these diseases will depend on the ability to express sufficient levels of AAV-mediated therapeutic protein in these cell types.

The ability to quickly characterize the transduction profiles of novel AAV vectors in biologically relevant, ocular cell lines would aid in developing vectors to treat retinal disease. There would be much to gain by having the ability to predict in vivo transduction efficiencies of the various capsid serotypes and capsid mutants through the use of a fast, high-throughput, in vitro assay. Such an assay would be valuable both at the ‘front end’ of gene therapy trials to quickly determine which serotype and/or promoter would potentially provide the level of transduction efficiency required for therapy at the proof-of-concept stage and at the ‘back end’ to develop appropriate cell-based assays to qualify clinical-grade vectors as part of a regulatory review before drug approval and future vector stability testing. The aforementioned retinal disorders highlight how cells that retain characteristics of RPE or cone photoreceptors would be particularly useful in such an assay.

The human retinal pigment epithelial cell line (ARPE19) is a spontaneously evolved, diploid human cell line, purified by selective trypsinizations of a primary RPE culture [9]. The cells have a normal karyotype, form polarized epithelial monolayers when cultured, and express several RPE-specific proteins [9]. They have been widely used as a model system for RPE, including studies evaluating key signaling pathways and responses to light damage and reactive oxygen species (ROS) [10-13]. The permeability characteristics of polarized ARPE19 monolayers improve with prolonged culturing and they are currently being used as a pharmacological and physiologic model to evaluate the barrier function of the outer retinal blood barrier [14,15]. A cell line of neural retina origin, 661W cone cells, was immortalized by the expression of SV40-T antigen under control of the human interphotoreceptor retinoid-binding protein (IRBP) promoter [16]. The 661W cells express cone but not rod photoreceptor markers, including red/green opsin and cone cyclic nucleotide gated channel subunits. They respond to light stimulation and undergo cell death when stressed by bright light [16,17]. They are increasingly being used as a surrogate to evaluate cone cell survival in response to oxidative stress/ROS in vitro [10,18]. In addition to the above characteristics, both cell lines are relatively easy to acquire and maintain. The purpose of this study was to use these biologically relevant ocular cell lines, ARPE19 and 661W (RPE and cone photoreceptor surrogates, respectively), to develop a high-throughput assay to quantify the transduction efficiencies of a variety of AAV vectors. We focused specifically on the relative transduction efficiencies of self-complementary vectors with capsids that were either unmodified or that contained single/multiple point mutations in which surface-exposed tyrosine residues were changed to phenylalanine (Y-F). We have shown that AAV vectors containing Y-F capsid mutations exhibit increased transduction efficiency in the retina relative to those with unmodified capsids [19]. Additionally, we have shown that an AAV capsid mutant vector was capable of restoring long-term retinal function and visual behavior and preventing retinal degeneration in an animal model of recessive retinitis pigmentosa, the *rd10* mouse model. This model is refractory to treatment with unmodified AAV vectors [20]. Here, we report the transduction efficiencies of each vector and examine the activity of several cell-specific and ubiquitous promoters with well characterized activity in ARPE19 and 661W cells, two biologically relevant ocular cell lines. Additionally, by comparing the results of transduction efficiencies for a subset of AAV2 Y-F mutants in mouse retina (i.e., in vivo) to our in vitro results, we evaluated the predictive utility of the assay.

## Methods

### Construction of AAV vectors

Self-complimentary rAAV serotypes 1, 2, 5, 8, 9, and their corresponding single and multiple tyrosine-to-phenylalanine (Y-F) mutants, all containing the ubiquitous, truncated chimeric CMV-chicken β-actin (smCBA) promoter [21] driving the mCherry reporter cDNA, were generated and purified by previously described methods [22,23]. Vectors were titered by quantitative real-time PCR and re-suspended in re-suspended in balanced salt solution (BSS; Alcon, Fort Worth, TX) [24].

### Cell culture

The ARPE19 (purchased from ATCC) and 661W cone cells (generously provided by Dr. Muayyad R. Al-Ubaidi, University of Oklahoma Health Sciences Center, Oklahoma City, OK) were routinely passaged by dissociation in 0.05% (w/v) trypsin and 0.02% (w/v) EDTA, followed by replating at a split ratio ranging from 1:3 to 1:5 in T75 flasks. Cells used for experiments were between passages 15 and 30. Cell morphology and growth rates were closely monitored at all steps to ensure cell line identity. The ARPE19 cells were maintained in culture medium consisting of Dulbecco’s modified Eagle’s medium (DMEM): Nutrient Mixture F12, 1:1 mixture with Hepes buffer containing 10% fetal bovine serum (FBS), 0.348% (w/v) additional sodium bicarbonate, 1% 200 mM L-glutamine, and 50 mg/ml Gentamicin [9]. The 661W cone cells were maintained in DMEM containing 10% FBS, 300 mg/l glutamine, 32 mg/l putrescine, 40 µl of β-mercaptoethanol, and 40 µg of hydrocortisone 21-hemisuccinate and progesterone. The media also contained penicillin (90 units/ml) and streptomycin (0.09 mg/ml) [16]. Cultures were incubated at 37 °C in 7% CO_2_.

### Infections

Cells were plated in 96-well plates and achieved 60%–70% confluency after overnight incubation. This confluency was achieved with seeding counts of 1.0×10^4^ - 3.0×10^4^ cells/well. Twenty-four hours post seeding, confluency was confirmed by bright-field microscopy (Nikon Diaphot). Next, AAV vectors were diluted in BSS and serum-free media to achieve the desired multiplicity of infection (MOI). MOI was defined as the number of genome containing vector particles per target cell.  Since recombinant AAV vectors have much lower physical to infectious particle ratios than 1:1, typically 1:20 to 1:100 [25], MOI in our case represents a theoretical upper limit to the number of infectious particles per target cell. Cells were rinsed in phosphate buffered saline (PBS) and the virus dilutions were applied at MOIs ranging from 100 to 10,000. After 1 h, 20% serum-containing media was added to the cells. Infected cells were incubated at 37 °C in 7% CO_2_ for 3 days, except where otherwise indicated. All infections were performed in triplicate. Each vector was used to infect 24 wells in total. Each “sample” was pooled from eight wells, making a final sample count of three. For long-term ARPE19 infection, cells were infected with scAAV2-smCBA-mCherry at an MOI of 10,000, as described above, and incubated for 14 days at 37 °C in 7.0% CO_2_. The 10% F-12 media was exchanged twice a week. Fluorescent images were taken at 3, 7, and 14 days post infection.

### Microscopy and fluorescence activated cell sorting analysis

Three days post infection, cells were observed using bright-field microscopy to ensure 100% confluency. Cells were then analyzed using fluorescent microscopy (Olympus IX70 Inverted Fluorescent Microscope equipped with a QImaging Retiga 4000R Camera with RGB-HM-5 Color Filter and QImaging QCapture Pro 6.0 software; QImaging Surrey, BC Canada). All images were taken at identical exposure settings (800 ms) and magnification (10×). mCherry expression was detected with a Semrock Bright Line TRITC-A filter with an excitation wavelength of 543 nm and an emission band pass of 553–633 nm. Finally, cells were dissociated with Accutase solution (MP Biomedicals, Solon, OH) and 10,000 cells per sample were counted and analyzed using a BD LSR II flow cytometer equipped with BD FACSDIVA 6.2 software (BD Biosciences, San Jose, CA). Uninfected control cells were also counted and analyzed to establish transduction efficiency baselines. Data was obtained from three samples for each vector. mCherry fluorescence was quantified with a PE-Texas-Red-A filter with an excitation wavelength of 532 nm and an emission band pass of 600–620 nm. The transduction efficiency of each AAV serotype and their respective Y-F capsid mutants were then calculated by multiplying the percentage of cells positive for mCherry by the mean fluorescence intensity [26]. The values of three samples for each vector were then averaged to obtain a final transduction efficiency value for each serotype.

### Promoter analysis

Serotype 2 self-complementary AAV vectors, containing the ‘humanized’ green fluorescent protein (GFP) reporter cDNA and one of four tissue-specific or ubiquitous promoters, were packaged and purified according to methods previously described [22-24]. Photoreceptor-specific promoters included the 501 bp mouse cone arrestin-3 (mCARpro) promoter [27] and the 471 bp mouse rod opsin (MOPS500) promoter [28]. The RPE-specific and ubiquitous promoters chosen were a 624 bp version of the vitelliform macular dystrophy/Best disease (VMD2) promoter [29] and smCBA [21], respectively. The ARPE19 and 661W cells were separately seeded in 24-well plates, achieving cell counts of 1.0×10^5^ cells/well. Twenty-four hours post seeding, the cells were exposed to each vector at an MOI of 10,000. Infections were performed as previously described in Method section. At 3 days post infection, immunostaining was performed according to previously described methods, with minor modifications [5]. Briefly, cells were fixed in 4% paraformaldehyde for 5 min, followed by three washes in 1× PBS (HyClone Laboratories, Inc. Logan, UT), a 15 min incubation in 0.5% Triton X-100 (diluted in 1× PBS), and a 30 min incubation in 1% bovine serum albumin (diluted in 1× PBS), all at room temperature. Cells were then incubated in primary antibody directed against GFP (A11122; rabbit polyclonal, Invitrogen) diluted 1:400 in 0.3% Triton X-100/1% BSA at room temperature for 3 h. Following primary incubation, cells were washed three times with PBS and incubated with secondary antibody (goat-anti rabbit IgG tagged with Alexa-Fluor 488, A11008; Invitrogen) diluted 1:500 in PBS for 1 h at room temperature. Lastly, cells were counterstained with 4’, 6’-diamino-2-phenylindole for 5 min at room temperature. Images were taken with the fluorescent scope, as previously described in the Methods section. A Semrock Bright Line GFP-3035B filter (Semrock, Inc., Rochester, NY) with an excitation wavelength of 472 nm and an emission band pass of 485 nm–555 nm was used to detect GFP expression. Images of cells infected with mCARpro-, MOPS500-, and VMD2-containing vectors were taken at identical exposure settings (800 ms) and magnification (10×), while 10× images of cells infected with smCBA-containing vectors were taken at an exposure time of 300 ms.

### In vivo analysis

All animals used in the study were maintained at the University of Florida Health Science Center in the animal care facilities under a 12 h:12 h light-dark environment and were handled in accordance with the Association for Research in Vision and Ophthalmology (ARVO) statement for the Use of Animals in Ophthalmic and Vision Research and the guidelines of the Institutional Animal Care and Use Committee at the University of Florida. Adult C57BL/6 mice were anesthetized with a mixture of ketamine (72 mg/kg)/xylazine (4 mg/kg) by intraperitoneal injection and were subretinally injected with 1 µl of vector at a titer of 1×10^13^ genome copies per ml, as previously described [30]. The AAV2-CBA-GFP triple mutant (Y444F+Y730F+Y500F) and AAV2-CBA-GFP sextuple mutant (Y444F+Y730F+Y500F+Y272F+Y704F+Y252F) used for this portion of the study have been previously described [31].

## Results

### Transduction efficiency of unmodified, self-complimentary AAV vectors in ARPE19 and 661W cells

**Figure 1:**
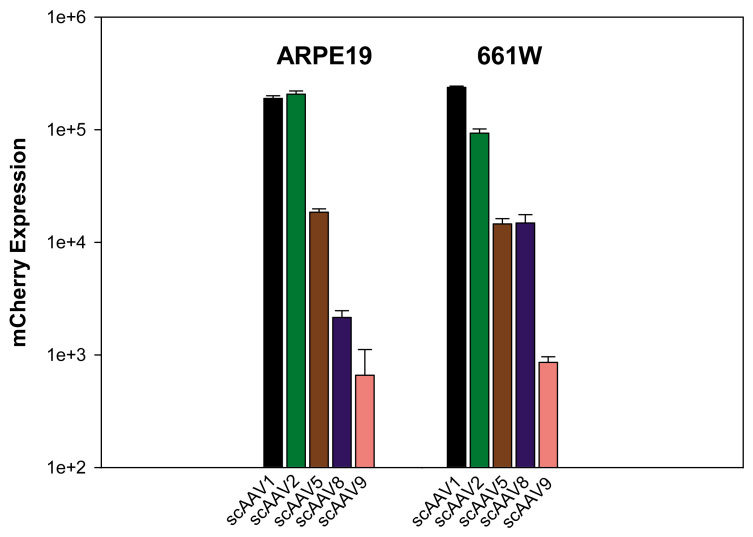
Transduction efficiency of unmodified scAAV vectors in ARPE19 and 661W cells. Cells were infected with scAAV1, 2, 5, 8, and 9 at a multiplicity of infection (MOI) of 10,000. mCherry expression is shown in arbitrary units calculated by multiplying the percentage of positive cells by the mean fluorescence intensity in each sample. Each value represents the average of three samples (eight pooled wells of a 24-well plate/sample), based on 10,000 counted cells.

The scAAV serotypes 1, 2, 5, 8, and 9 infected in both ARPE19 and 661W cells at MOIs ranging from 100 to 10,000 and their transduction efficiencies (as measured by mCherry expression) were analyzed three days later by flow cytometry. Here, we present data generated from cells infected only at an MOI of 10,000 because it is representative of trends seen across all MOIs. Our results indicate scAAV1 and scAAV2 had the highest transduction efficiency in both cell lines (Figure 1). In ARPE19 cells, scAAV1 and scAAV2 had very similar transduction efficiencies. scAAV5 and scAAV8 had substantially lower transduction efficiencies than scAAV1 and scAAV2 (10-fold and 100 fold lower, respectively). In 661W cells, scAAV1 had 2.5 fold higher transduction efficiency than scAAV2. scAAV5 and scAAV8 had similar transduction efficiencies, both sevenfold lower than scAAV2. scAAV9 had the lowest transduction efficiency in both cell lines.

### Transduction efficiency of modified scAAV2 vectors in ARPE19 and 661W cells

**Figure 2:**
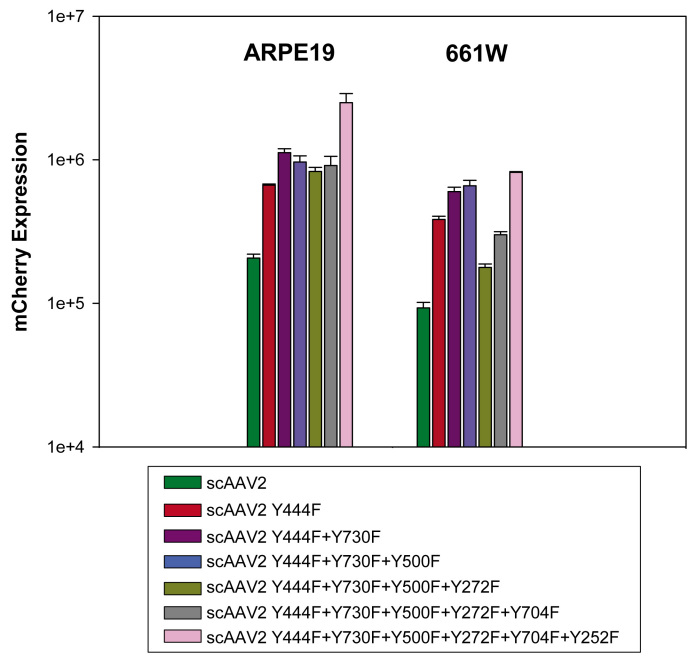
Transduction efficiency of unmodified scAAV2 and its single/multiple Y-F capsid mutant vectors. Cells were infected with unmodified scAAV2, scAAV2 single mutant (Y444F), double mutant (Y444F+Y730F), triple mutant (Y444F+Y730F+Y500F), quadruple mutant (Y444F+Y730F+Y500F+Y272F), pentuple mutant (Y444F+Y730F+Y500F+Y272F+Y704F), and sextuple mutant (Y444F+Y730F+Y500F+Y272F+Y704F+Y252F) at a multiplicity of infection (MOI) of 10,000. mCherry expression is shown in arbitrary units calculated by multiplying the percentage of positive cells by the mean fluorescence intensity in each sample. Each value represents the average of three samples (eight pooled wells of a 24-well plate/sample), based on 10,000 counted cells.

We have shown that serotype 2 AAV vectors containing multiple combinations of Y-F capsid mutations exhibit increased transduction efficiency relative to unmodified AAV2 vectors in adult mouse retinas [19]. Here, we evaluate whether the same pattern can be found in the ARPE19 and 661W ocular cell lines. Vectors tested included the scAAV2 single mutant (Y444F), double mutant (Y444F+Y730F), triple mutant (Y444F+Y730F+Y500F), quadruple mutant (Y444F+Y730F+Y500F+Y272F), pentuple mutant (Y444F+Y730F+Y500F+Y272F+Y704F), and sextuple mutant (Y444F+Y730F+Y500F+Y272F+Y704F+Y252F). Infections were performed as previously described. We found that all scAAV2 Y-F capsid mutants have higher transduction efficiencies than unmodified scAAV2 (Figure 2). Of all the vectors, the scAAV2 sextuple mutant had the highest transduction efficiency in both cell lines; its transduction efficiency was 12.5 fold and 9 fold higher than unmodified scAAV2 in ARPE19 and 661W cells, respectively.

### In vivo confirmation of in vitro transduction patterns for selected AAV2 Y-F mutants

**Figure 3:**
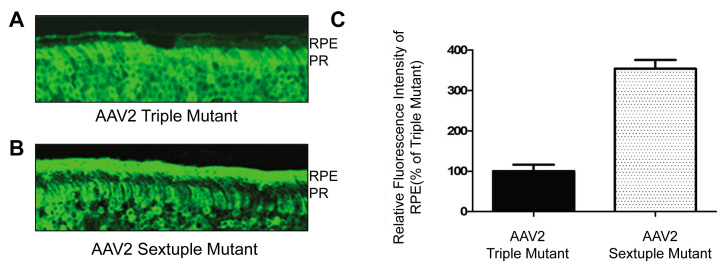
In vivo transduction pattern of AAV2 triple and sextuple Y-F capsid mutants. Green fluorescent protein (GFP) expression in retinal sections of adult mice at one month following subretinal delivery of (**A**) AAV2 triple mutant (Y444F+Y730F+Y500F) or (**B**) sextuple mutant (Y444F+Y730F+Y500F+Y272F+Y704F+Y252F) and (**C**) a comparison of the relative GFP fluorescence intensity in the retinal pigment epithelium (RPE) layer of retinas treated with the triple and sextuple AAV2 vectors, respectively. Values indicate percent GFP intensity relative to treatment with AAV2 triple mutant vector. All pictures were evaluated using Image J software. The data are shown as the mean±SEM; n=3 for each group (p<0.0001). PR represents photoreceptor layer.

In Figure 2, the scAAV2 sextuple Y-F mutant was shown to be the most efficient multiple Y-F mutant in ARPE19 cells, approximately threefold higher than the triple Y-F mutant, whereas in 661W cells the triple and sextuple Y-F mutants were equally efficient. Based on this observation, we would predict that, in vivo, the sextuple mutant would show significantly higher transduction efficiency in RPE that the triple mutant. Figure 3 demonstrates that, when delivered to the subretinal space, the AAV2 sextuple mutant mediates more efficient transduction of RPE than a matched (same construct and vector titer) AAV2 triple mutant and that when compared, the relative efficiencies of these vectors in vitro and in vivo (Figure 2 and Figure 3, respectively) are well correlated.

### Fluorescent microscopy analysis

**Figure 4:**
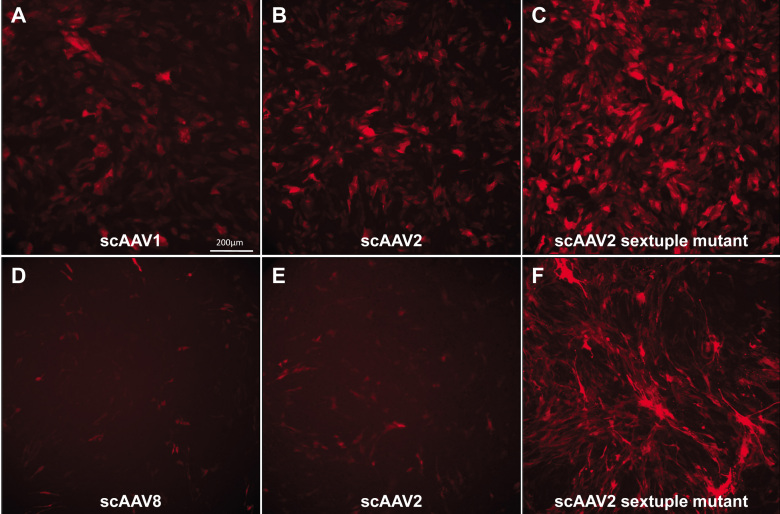
scAAV-mediated mCherry expression in human retinal pigment epithelial (ARPE19) and 661W cone photoreceptor cells, at three days post infection. The top row consists of representative images of mCherry expression in ARPE19 cells infected with (**A**) scAAV1, (**B**) scAAV2, and (**C**) scAAV2 sextuple mutants at a multiplicity of infection (MOI) of 10,000. The bottom row consists of representative images of mCherry expression in 661W cells infected with (**D**) scAAV8, (**E**) scAAV2, and (**F**) scAAV2 sextuple mutants at an MOI of 10,000. All 10× images were taken with identical exposure times (800 ms). The scale bar in A=200 µm.

In addition to FACS analysis, fluorescent images were taken of all infections at all MOIs at 3 days post infection. Representative images shown in Figure 4 are of ARPE19 or 661W cells infected at an MOI of 10,000. Because scAAV1 promoted good transduction efficiency in ARPE19 cells (Figure 1) and is known to target RPE in vivo following subretinal injection [32], we present images for this serotype in ARPE19 cells (Figure 4A). Similarly, because scAAV8 was shown to efficiently transduce 661W cells (Figure 1) and because this serotype is known to target photoreceptor cells in vivo following subretinal injection [32], we present images for this serotype in 661W cells (Figure 4D). In addition, images of scAAV2 and its respective sextuple mutant (shown to be the most efficient of all capsid mutants tested via FACS analysis in Figure 2) are shown in each cell line. While other serotypes generated results with the more sensitive FACS analysis, their transduction efficiencies were too low to produce images with discernable fluorescence in this assay. Consistent with the results of our FACS analysis, fluorescent images show that scAAV1 and scAAV2 had high and relatively similar transduction efficiencies in ARPE19 cells (Figure 4A, B). An apparent increase in transduction efficiency was observed in cells infected with the scAAV2 sextuple mutant; there were more fluorescent cells and a stronger overall mCherry signal within each cell (Figure 4C). The FACS analysis also showed that even with an ~7 fold decrease in transduction efficiency compared to scAAV2 (Figure 1), an infection with scAAV8 resulted in visible mCherry expression in 661W cells (Figure 4D,E). An increase in transduction efficiency was also seen with the scAAV2 sextuple infection in 661W cells (Figure 4F). These results indicate that vectors ~7 fold less efficient than unmodified scAAV2 may still be visualized via fluorescent microscopy.

### Transduction efficiency of scAAV1, scAAV8, and their single Y-F capsid mutants

**Figure 5:**
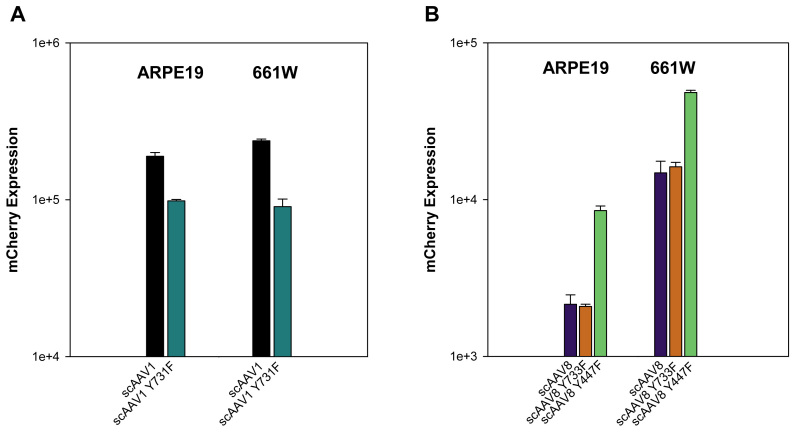
Transduction efficiency of unmodified scAAV1, scAAV8, and their respective single Y-F capsid mutant vectors. Transduction efficiency of scAAV1 and scAAV1 Y731F (**A**), scAAV8, scAAV8 Y733F, and scAAV8 Y447F (**B**) in ARPE19 and 661W cells measured by mCherry-mediated and fluorescence-activated cell sorting (FACS) analysis. Data represents infections at a multiplicity of infection (MOI) of 10,000. mCherry expression is shown in arbitrary units calculated by multiplying the percentage of positive cells by the mean fluorescence intensity in each sample. Each value represents the average of three samples (eight pooled wells of a 24-well plate/sample), based on 10,000 counted cells.

Because of the known tropism of scAAV1 and scAAV8 for RPE and photoreceptors in vivo [32], respectively, an investigation of the transduction efficiencies of their single Y-F capsid mutants was performed. Vectors infected in both cell lines included scAAV1, scAAV1 Y731F, scAAV8, scAAV8 Y733F, and scAAV8 Y447F. Unmodified scAAV1 had relatively high transduction efficiency in both ARPE19 and 661W cells (Figure 1). However, the respective single capsid mutant was less efficient in both cell lines (Figure 5A). The single capsid mutant, scAAV8 Y447F, demonstrated a fourfold and threefold increase in transduction efficiency over scAAV8 in ARPE19 and 661W cells, respectively. However, no appreciable increase in efficiency was noted for scAAV8Y733F over scAAV8 in either cell line.

### ARPE19 cells can withstand lengthy post infection incubation times

**Figure 6:**
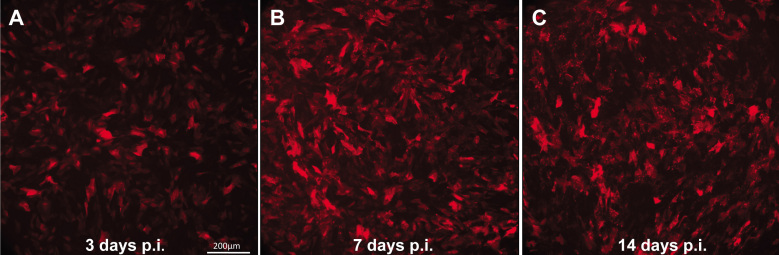
Human retinal pigment epithelial cell line (ARPE19) cells remain viable upon lengthy post infection incubation. mCherry expression in ARPE19 cells infected with scAAV2-smCBA-mCherry at an MOI of 10,000 (**A**) 3 days, (**B**) 7 days, and (**C**) 14 days post infection. All 10× images were taken at identical exposure settings (800 ms). The scale bar in **A**=200 µm.

ARPE19 cells grow as a distinct monolayer capable of living for months without being passaged if fed regularly with serum-containing medium (data not shown). We sought to determine whether ARPE19 cells would remain healthy for up to 14 days after infection with AAV, the idea being that if the cells remained healthy and continued to accumulate gene product as a function of time, they could be useful in developing bioassays requiring maximal viral-mediated protein expression. In contrast to ARPE19 cells, we noted that 661W cells became overcrowded after a few days (data not shown) and required regular passaging. For this reason, 661W cells were not analyzed in this manner. Figure 6 demonstrates the long-term viability of ARPE19 cells infected with scAAV2-smCBA-mCherry. An increase in gene product can clearly be seen between 3 days and 7 days post infection (Figure 6A,B). Cells remained healthy at 14 days post infection, but mCherry expression appeared only marginally increased relative to that seen at 7 days post infection (Figure 6B,C). This suggests that maximal AAV-mediated transgene expression was reached somewhere between 7 and 14 days post infection.

### Promoter analysis

**Figure 7:**
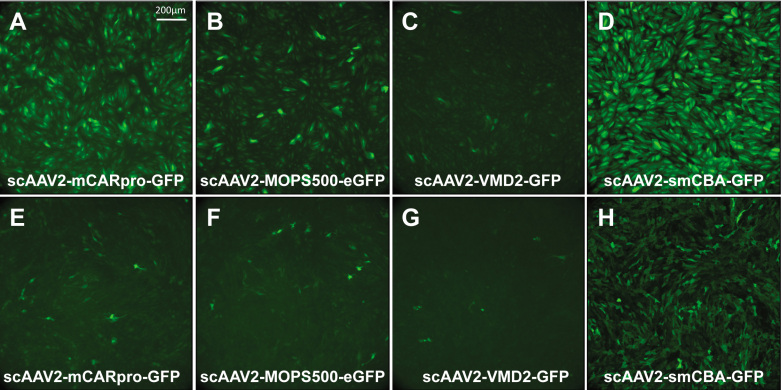
Retina-specific promoters are capable of transducing both human retinal pigment epithelial cell line (ARPE19) and 661W cells. Representative images of green fluorescent protein (GFP) expression in ARPE19 cells at 3 days post infection with AAV2 (**A**) sc-mCARpro-GFP, (**B**) sc-MOPS500-eGFP, (**C**) sc-VMD2-GFP, and (**D**) sc-smCBA-GFP. Representative images of GFP expression in 661W cells at 3 days post infection with AAV2 (**E**) sc-mCARpro-GFP, (**F**) sc-MOPS500-eGFP, (**G**) sc-VMD2-GFP, and (**H**) sc-smCBA-GFP. All infections were done at a multiplicity of infection (MOI) of 10,000. 10× images **A**-**C** and **E**-**G** were taken at an ET of 800 ms, whereas images **D** and **H** were taken at an ET of 300 ms. The scale bar in **A**=200 µm.

We sought to evaluate a representative selection of retina-specific promoters already successfully used in conjunction with AAV in proof-of-concept gene therapy experiments. scAAV2 vectors expressing green fluorescent protein (GFP) under the control of either mCARpro (cone- targeting), MOPS500 (rod-targeting), VMD2 (RPE-targeting), or smCBA (ubiquitous) were infected in both ARPE19 and 661W cells at an MOI of 10,000. At 3 days post infection, cells were immunostained and imaged. In ARPE19 cells, GFP expression was apparent in infections with all four promoters (Figure 7A-D). The ubiquitous smCBA promoter appeared to drive the highest levels of GFP expression, followed by mCARpro, MOPS500, and VMD2. GFP expression was also seen in all four infections in 661W cells (Figure 7E-H). Transduction was most evident with the ubiquitous smCBA promoter followed by mCARpro and MOPS500, which showed relatively equal GFP expression. The VMD2 promoter appeared to be the least effective in both cell lines (Figure 7C,G).

## Discussion

Here, we report the relative transduction efficiencies of several well characterized AAV serotypes and their respective single/multiple tyrosine capsid mutants in two biologically relevant ocular cell lines, ARPE19 (RPE) and 661W (cone photoreceptor) cells. In addition, we examine the activity of rod-specific, cone-specific, RPE-specific, and ubiquitous promoters when delivered by scAAV2 to both lines. We chose ARPE19 (human origin) and 661W cells (mouse origin) because: 1) they are derived from two retinal tissues, RPE and cone photoreceptors, that are well established as viable targets of AAV-mediated gene therapy, 2) both cell lines are increasingly used to model important physiologic processes intrinsic to RPE and cone photoreceptors, and 3) both are easy to maintain in culture and therefore adaptable to a high through-put application. Non-ocular cell lines, such as human embryonic kidney (HEK293) and HeLa cells, are typically used to assay AAV vectors in vitro because of their availability. However, with the exception of AAV2, many serotypes transduce these non-ocular cell lines very inefficiently. In addition, non-ocular HEK293 and HeLa cells are often incapable of supporting photoreceptor-specific or RPE-specific protein expression (data not shown). Here, we report that scAAV serotypes 1, 2, 5, and 8, as well as scAAV1, scAAV2, and scAAV8 capsid mutants, were capable of transducing RPE and cone photoreceptor cell lines in vitro. In addition, we show that photoreceptor-specific, RPE-specific, and ubiquitous promoters are active in both cell lines. Our results have implications for investigators seeking to optimize AAV-based gene therapy vectors to treat retinal disease and/or develop quick assays for qualifying clinical-grade vectors.

The scAAV serotypes, 1, 2, and 5 transduce both ARPE19 and 661W cells relatively well. The efficient transduction of both cell lines by scAAV1 is not surprising given the reports of AAV1’s preference for RPE and photoreceptors in vivo [32,33]. Likewise, scAAV8 transduces 661W cells more effectively than ARPE19s.This result is consistent with reports that AAV8 primarily transduces photoreceptors and some RPE cells in vivo [32]. In fact, recent reports show that AAV8 has been used to drive therapeutic expression in and restore function to photoreceptors in several models of inherited retinal degeneration, including Leber congenital amaurosis (AIPL1 and RPGRIP forms) [34-36] and X-linked juvenile retinoschisis [37]. Taken together, these results indicate a degree of similarity between the patterns of transduction expected of AAV1 and AAV8 in vitro and in vivo. Likewise, we show that scAAV5 efficiently transduces both cell lines, a result also consistent with AAV5′s preference for photoreceptors and RPE in vivo [33]. In comparison, despite its ability to efficiently transduce both RPE and photoreceptors in the murine retina [38-40], scAAV9 inefficiently transduces ARPE19 and 661W cells.

Mutations of surface-exposed AAV capsid tyrosine residues increase vector transduction kinetics and efficiency relative to matched, unmodified vectors [41]. It is thought that by replacing tyrosine residues with phenylalanine, these vectors evade phosphorylation and subsequent ubiquitination, thus preventing proteasome-mediated vector degradation and facilitating intracellular trafficking of the vector genome to the nucleus [41].

Accordingly, we show here that scAAV2 vectors with capsids containing as few as one and as many as six Y-F mutations transduce ARPE19 and 661W cells more effectively than their unmodified counterpart, scAAV2. The sextuple mutant (scAAV2 Y444F+Y730F+Y500F+Y272F+Y704F+Y252F) exhibited the highest transduction efficiency in both cell lines. However, it is interesting to note that the relationship between the transduction efficiency of scAAV2 vectors and the amount of Y-F mutations present on their capsids is not linear. While still more efficient that standard scAAV2, we found that the transduction efficiency of the quadruple mutant (scAAV2 Y444F+Y730F+Y500F+Y272F) in both cell lines was reduced relative to vectors containing fewer mutations. The addition of the Y272F residue (going from triple to quadruple mutant) had a substantial negative effect on transduction efficiency in the 661W cells. This pattern is consistent with what was observed in a similar study, in which multiple scAAV2 Y-F mutants were evaluated in HeLa cells and murine hepatocytes [42]. The mechanism underlying this Y272F-associated reduction is unclear. It is possible that replacing a hydrophilic tyrosine residue at position 272 of the capsid protein with a hydrophobic phenylalanine sufficiently alters surface configuration and hinders binding, uptake, or retention of vectors to/in its target cell. It would be interesting to evaluate whether transduction efficiencies of the quadruple, pentuple, and sextuple mutants would be further enhanced by removing the Y272F mutation. Our observations of transduction efficiencies in 661W cells are consistent with our previous report of AAV2 multiple Y-F mutant-mediated transduction in mouse retina following subretinal injection of vectors [31]. In vivo, the triple mutant was most efficiently expressed in photoreceptors [31]. Here, we show the triple and sextuple mutants were the most efficient at transducing 661W cells. In both cases, there was a drop off in efficiency when moving from the triple to the quadruple mutant. However, the comparison is complicated by the fact that when delivered to the subretinal space, the vector can also directly access RPE, whereas in vitro, the vector is exposed to only one cell type. Furthermore, our in vitro data indicates the sextuple mutant transduces ARPE19 cells much more effectively than the triple mutant (~3 fold better). We therefore investigated the reproducibility of this result, in vivo, by examining whether the sextuple mutant would show improved transduction efficiency in RPE relative to the triple mutant when delivered to the subretinal space. As predicted, the relative transduction efficiencies for the sextuple and triple mutants in vivo (RPE) and in vitro (ARPE19) were consistent. The increased transduction efficiencies for AAV2 Y-F mutants is not a straightforward story, as they show enhanced transduction of retinal layers distal to the injection site [31]. In particular, when delivered to the vitreous, the AAV2 Y-F quadruple, pentuple, sextuple, and septuple mutants transduced all retinal layers, including photoreceptors and in some cases, RPE [31]. This potential for “penetration” cannot be modeled in vitro, highlighting one limitation of cell-based assays.

Unlike the point mutations in scAAV2 capsids, which generally increased transduction efficiency relative to the unmodified vector, certain single point mutations on the scAAV1 and scAAV8 capsids examined here did not increase the transduction efficiency relative to their unmodified counterparts. In fact, scAAV1 Y731F was less efficient than unmodified scAAV1 in both ARPE19 and 661W cells. Likewise, the single Y733F mutation did not markedly alter the transduction efficiency of scAAV8. It is worth noting that 731, 733, and 730 correspond to the equivalent capsid residues of scAAV1, scAAV8, and scAAV2, respectively [43]. Is it possible that inhibiting phosphorylation of this specific residue may be relatively ineffective at increasing transduction efficiencies of any AAV serotype? While we did not test the corresponding single mutant in this study, scAAV2 Y730F has shown reduced efficiency in CD34^+^ stem cells [44]. However, scAAV2 Y730F exhibited improved transduction relative to unmodified scAAV2 in HeLa cells and murine hepatocytes in vitro, as well as murine retina and hepatocytes in vivo [19,42]. We have shown that scAAV8 containing the corresponding single Y733F capsid mutation mediates more rapid transgene expression than its unmodified counterpart, scAAV8, in murine retina [19]. In addition, we recently demonstrated that the AAV8 Y733F-vectored transgene restored vision to a model of early-onset recessive retinitis pigmentosa, the *rd10* mouse model. This model is refractory to long-term therapy with unmodified AAV8 [20]. As discussed, in light of the novel characteristics exhibited by AAV2 multiple Y-F mutants, these results indicate that cell lines in culture are not a perfect substitute for characterizing in vivo vector behavior. There are additional factors at play that affect transduction efficiency, which cell-based assays will struggle to appropriately model. These include physical barriers to vectors, such as the inner limiting membrane, which restricts AAV-mediated transduction of the outer retina following delivery of a vector to the vitreous cavity and which varies in degree from species to species [45],  immunological barriers, either pre-existing immunity to AAV capsid protein or cell-mediated immune response to AAV capsid and/or transgenes, which can block AAV-mediated gene expression, again the degree to which varies widely among species [46]. The developmental state and/or condition of the target tissue, the observations that AAV vectors exhibit different transduction patterns when delivered to developing as opposed to fully differentiated retina [47] and show enhanced transduction characteristics in degenerating retina [48], highlight potential changes in cell surface receptors as well as structural changes that ultimately effect AAV transduction. However, as indicated by the example of our assay to correctly make a prediction of in vivo transduction, in vitro models do have utility. Given the immense number of capsid variants likely to be available in the future, in vitro assays will be a useful first step in characterizing novel variants and will provide important clues for vector design strategies. For instance, our results show that scAAV8 Y447F exhibited marked improvements in transduction efficiency relative to unmodified scAAV8 in both cell lines. The same pattern was seen with the corresponding single mutant [43], scAAV2 Y444F. This result, in addition to the observations about AAV8 Y733F in vivo, suggests that combining the Y444F and Y733F mutations may have an additive effect. These and other combinations of Y-F mutations are currently being made to test this idea.

The ARPE19 and 661W cells also proved useful for testing the activity of photoreceptor-specific, RPE-specific, and ubiquitous promoters. This is an important finding given the need to characterize vectors for retinal disease in cell-based assays and that cell lines typically used for such in vitro vector characterization (HEK293, HeLa) often do not support expression of retinal-specific proteins. Here, we show that cone-specific (mCARpro), rod-specific (MOPS500), RPE-specific (VMD2), and ubiquitous (smCBA) promoters were active in both cell lines. All promoters qualitatively appeared to be more active in ARPE19 cells than in 661Ws. Nevertheless, GFP expression from scAAV2 vectors containing each promoter could be visualized in both lines. As expected, the strong smCBA promoter drove the most robust reporter expression, as it does in vivo. The recently characterized ‘mCARpro’ promoter also drove robust expression in ARPE19 cells. This result is similar to what we have seen and others have reported for mouse retinas [31,49]. Interestingly, we found the VMD2 promoter, which has demonstrated RPE-specificity in vivo [50], reduced activity in the ARPE19 cells relative to the two photoreceptor targeting promoters. Overall, the pattern of expression in the 661W cells was more consistent with what would be predicted based on in vivo characteristics of the promoters, with the cone-targeting mCARpro performing better than the rod-targeting MOPS500s and better still than the VMD2 promoter.

In addition to their utility for screening a variety of unmodified and modified scAAV serotypes and various retina-specific and ubiquitous promoters, we also show that ARPE19 cells maintain viability upon long incubation times. Because many cells, including 661Ws, grow without contact inhibition in culture until they become overcrowded and die, assays for vector-mediated transgene expression must be done within a few days of infection. On the other hand, ARPE19 cells grow in culture until they successfully form a healthy monolayer that persists for months. Here, we show that ARPE19 cells can be transduced with an AAV vector and assayed as late as two weeks post infection (the latest time point evaluated), a length of time not afforded by traditional cell lines. This may be critical for investigators seeking to evaluate expression of AAV-vectored proteins that require longer incubation times to reach detectable levels of expression. Additionally, those interested in evaluating the long-term effects of AAV-mediated transgene expression or simply evaluating the long-term effects of AAV transduction alone on cellular physiology may consider using ARPE19 cells.

Using FACS analysis, we have developed a high-throughput assay for quantifying the relative transduction efficiencies of a variety of self-complementary AAV vectors in two ocular cell lines, RPE-derived, ARPE19, and cone photoreceptor-derived 661W. Both lines were used to compare the efficiency of scAAV vectors that have either unmodified capsids or capsids containing single/multiple tyrosine-phenylalanine (Y-F) mutations. Quantification with FACS was supplemented with qualitative analysis of vector performance in each cell line via imaging of reporter protein (mCherry) expression. With limited exceptions, the transduction profiles of vectors transduced into ARPE19 and 661W cells are consistent with reports of their behavior in vivo. In addition, we have shown that both ocular cell lines support the activity of rod-specific, cone-specific, RPE-specific, and ubiquitous promoters, albeit to different degrees. The ARPE19 cells remained viable for at least two weeks post infection, a factor that makes this cell line useful for long-term in vitro assays. The recent success of several ongoing clinical trials for RPE65-Leber congenital amaurosis-2 as well as proof-of-concept studies involving mice, dogs, and monkeys have established that AAV-mediated gene replacement is a feasible strategy for treating a wide variety of RPE and photoreceptor-specific disorders. Our results suggest that this rapid cell-based assay using two biologically relevant ocular cell lines will prove a useful adjunct in screening and/or optimizing vector constructs and can be used in conjunction with small molecules/drugs aimed at increasing AAV transduction efficiency.
